# Recent Advances and Therapeutic Options in Lambert-Eaton Myasthenic Syndrome

**DOI:** 10.7759/cureus.5450

**Published:** 2019-08-21

**Authors:** Arsalan Anwar, Sidra Saleem, Mirza Fawad Ahmed, Sara Ashraf, Sameen Ashraf

**Affiliations:** 1 Neurology, University Hospitals Cleveland Medical Center, Cleveland, USA; 2 Neurology, University of Toledo, Toledo, USA; 3 Internal Medicine, Lahore Medical and Dental College, Lahore, PAK; 4 Internal Medicine, Sharif Medical and Dental College, Lahore, PAK; 5 Internal Medicine, Dow Medical College and Civil Hospital Karachi, Karachi, PAK

**Keywords:** lambert - eaton myasthenic syndrome, therapy, prognosis, pathophysiology

## Abstract

Lambert-Eaton Myasthenic Syndrome (LEMS) is an autoimmune-mediated neurological disorder that manifests as muscle fatigue, diminished tendon reflexes, with symptoms of cholinergic overactivity. It can be associated with certain neoplastic conditions, the most common being small cell lung carcinoma (SCLC). The basic pathophysiology involved is antibody-mediated targeting of voltage-gated calcium channels (VGCC), which decreases the release of acetylcholine in the synaptic junction. Multiple treatment options have been introduced in the past and, recently, a new drug, amifampridine, has been approved by the Food and Drug Administration (FDA) for the treatment of weakness associated with these patients. We summarize this newly introduced drug with a brief description of other treatment options available.

## Introduction and background

A revolutionary article was published in the Journal of the American Medical Association (JAMA) in 1956, briefing about the syndrome with the neuromuscular transmission defect discovered by Eaton and Lambert, which was the basis for the coined name of the disease, Lambert-Eaton Myasthenic Syndrome (LEMS) [1]. LEMS is an autoimmune disorder of the neuromuscular junction caused by antibodies produced against the voltage-gated calcium channels (VGCC) on the presynaptic nerve terminals, thus inhibiting the release of the neurotransmitter acetylcholine (ACh) [2]. The clinical manifestation of the disease is muscle fatigue, which principally affects the proximal parts of extremities. The tendon reflexes are absent or diminished in these patients [3]. LEMS is also accompanied by symptoms that are representative of cholinergic dysautonomias such as decreased salivation, sweating, constipation, and impotence. Oculobulbar involvement, presenting as ptosis or diplopia, is seen more in myasthenia gravis (MG) as compared to LEMS [4]. LEMS is classified as paraneoplastic or idiopathic. A large fraction of LEMS cases have an underlying tumor, primarily small cell lung carcinoma (SCLC). The occurrence of MG is 46 times more than LEMS. LEMS has a male predominance in 60%-75% of patients in contrast to MG where most cases are seen in females. The age of onset in patients with non-paraneoplastic LEMS is the same as in MG, which is usually around 35 years of age. In contradiction, paraneoplastic LEMS peaks at around 58 years. Seventy-three percent of SCLC individuals are also confirmed as having LEMS [5].

## Review

Etiology

LEMS is elicited by auto-antibodies that form against the VGCC present in the cell membrane of neurons. These anti-VGCC antibodies are highly sensitive, as they can be detected in 85% of affected individuals. Most frequently, the VGCC autoantibodies detected in such patients' serum are formed against the alpha1 subunit of presynaptic receptors and bind with the alpha1 subunit or, rarely, the beta3 subunit. Therefore, various parts of the presynaptic VGCC complex are potential targets for antibodies [6-7].

It is also reported that non-paraneoplastic LEMS patients are associated with underlying immune-mediated diseases. Wirtz et al. concluded that 27% of non-paraneoplastic LEMS patients and 11% of paraneoplastic LEMS had underlying immune disorders, including type 1 diabetes and thyroid disease [8]. Titulaer et al. showed, in a small case series of paraneoplastic LEMS patients, a persistent affiliation with human leukocyte antigen (HLA)-B8 in class l and HLA-DQ2 and HLA-DR3 in class ll. Around 65% of non-paraneoplastic LEMS patients were found to be HLA-B8 positive, and 50% were HLA-A1 positive while the same frequency existed for HLA-DQ2 and HLA-DR3 [9].

In both the paraneoplastic and idiopathic forms of LEMS, clinical symptoms are due to an antibody-mediated reduction of VGCC in the presynaptic terminal of the neuromuscular junction (NMJ). Reduction in VGCC leads to a decrease in Ca2+ influx, which is required for presynaptic vesicle fusion and neurotransmitter release. This neurotransmitter, acetylcholine (Ach), is required for postsynaptic muscle contraction. Although in LEMS, NMJ compensates for VGCC, but this compensation is not sufficient to restore the normal amount of neurotransmitter release and thus muscle contraction [10].

Diagnosis

LEMS is first suspected based on clinical signs and symptoms showing the classic triad of proximal muscle weakness, decreased tendon reflexes, and autonomic dysfunction [11]. The clinical findings need to be confirmed by different electrophysiological studies, such as electromyography (EMG) and nerve conduction studies (NCS). Motor and sensory nerve conduction studies show that the compound muscle action potential (CMAP) amplitude of resting muscle in LEMS patients is lower than the standard [12]. The choice of test for diagnosis is repetitive nerve stimulation (RNS) where a low-frequency stimulation of 2-5 Hz shows a decremental response [8]. A reproducible increase in the CMAP amplitude of 100% or more, with a high-frequency stimulation of 50 Hz (post-activation stimulation), or after vigorous stimulation of muscle for 10s (post-exercise stimulation) confirms LEMS [13]. Needle EMG shows erratic changes in motor unit action potential as low and short during the voluntary action potential. This can be followed by single-fiber EMG measurements of jitter. The increase in jittering shows transmission-blocking and corresponds with the severity of the disease [14].

A blood test to detect antibodies against VGCC on the nerve side of the NMJ is present in 85%-90% of patients with LEMS [2]. The test alone is not confirmatory of the disease, but it is a sensitive test along with clinical signs. The presence of antibodies to VGCC does not correspond with the severity of the disease, but as the patient is started on immunosuppressive therapy, sensitivity to this test is decreased, as antibody production is reduced, and is effective when performed before the start of treatment [13]. VGCC has two types of antibodies targeted towards them and P/Q type antibodies are most commonly seen in LEMS but a very small percentage has also shown type-N antibodies with an increased incidence of SCLC in them [15].

Treatment options

There are multiple treatment options available for the symptomatic treatment of LEMS and the most effective ones are those that increase acetylcholine release from the presynaptic membranes. If LEMS and malignancy are present, the primary concern is cancer therapy as successful tumor therapy in LEMS patients also helps reduce the symptoms of LEMS [16-17]. The treatment option can be characterized depending upon its therapeutic impact on improving weakness in patients.

In patients with mild weakness, regular follow-up is advised and patients are treated if any concomitant malignancy is present [18]. The following are some drug options explained along with recent advances to treat moderate to severe and refractory weakness.

Recent advancement

Amifampridine

The main therapeutic focus in LEMS is to improve neuromuscular transmission. Many symptomatic treatments have been experimented in the past few decades, including pyridostigmine, guanidine, 4-aminopyridine, and 3,4-Diaminopyridine(3,4-DAP), with the last one proving to be the most efficacious [19]. 3,4-DAP binds to the VGCC, causing them to remain open for a longer duration by blocking presynaptic potassium channels. These potassium channels prolong the depolarization at the nerve endings, thus increasing the calcium influx with the subsequent release of ACh from motor nerve terminals improving LEMS-related muscle weakness [20].

Many trials were conducted in the recent past that have successfully shown the drug’s efficacy and improvement in the Quantitative Myasthenia Gravis (QMG) score. The side effects of the normal therapeutic range are mild, including perioral and extremity paresthesias, nausea, vomiting, and elevated liver enzymes. The side effects of more severity, such as seizures, have a low risk of occurrence because of the drugs low penetration in the CNS, but as it is dose-dependent, it can occur at doses greater than 100 mg per day. 3,4-DAP was first approved in Europe in December 2009 and later, in 2010, it was recommended as a first-line symptomatic treatment for LEMS [21]. Amifampridine phosphate is the salt form of the 3-4-DAP base and it is more stable [22]. The starting dose of amifampridine prescribed for adults is 15-30 mg orally three times a day. The maximum approved daily dose is 80 mg a day. The US Foods and Drug Administration (FDA) approved the use of amifampridine in November 2018 for adults (Firdapse) [23].

Table 1 summarizes the trials showing the efficacy of amifampridine in patients suffering from LEMS.

**Table 1:** Clinical trials (amifampridine)

Year/Study	Description (methodology)	Result
Wirtz et al. [19], 2008, Netherlands.	9 patients underwent four treatment sessions for two successive days with 3,4- DAP, where RNS and muscle strength were recorded every time during drug administration to note any changes.	Combination therapy with 3-4-DAP and pyridostigmine was not statistically significant than 3-4-DAP alone.
Sanders et al. [24], 1999, North Carolina, USA.	26 patients were divided into two groups, one group with 12 patients was taking 3-4-DAP and another group was on placebo. QMG score and secondarily CMAP amplitude was measured in both groups.	The QMG score and secondary CAMP amplitude showed improvement with minimum side effects in the treatment group.
McEvoy et al. [25] 1989, Minnesota, USA.	12 patients were given open-label 3,4-DAP orally 25mg four times a day for the first 8 days and later assigned randomly for either drug or placebo on the next 3 days.	Neurologic disability scores decreased with increased muscle strength and CMAP amplitude. Side effect such as seizure is dose-dependent.
Oh SJ et al. [26], 2016, Alabama, USA.	A ‘withdrawal trial’ was carried out in 4 parts. In part 1 optimal tolerated dose of 3,4-DAP was given which was then tapered off and replaced with placebo, in randomly selected patients, in part 2. In part 3 the established dosage was then administered for seven additional days with some patients taking part in safety assessment up to two years in part 4.	Primary efficacy, QMG, and SGI scores were markedly improved in Day 8 and 14 of the studies, hence proving the efficacy of Amifampridine use.
Oh SJ et al. [27], 2009, Alabama, USA.	7 patients, randomly assigned placebo and DAP, had baseline measurements of subjective scores and muscle strength, QMG score, RNS, and SFEMG.	DAP changes the difference in values from baseline was statistically significant, unlike placebo change.
Keogh et al. [28], 2011. Tyne, UK.	2 placebo-controlled trials consisting of 38 patients consuming 3,4-DAP. A third crossover trial in 9 patients receiving IV immunoglobulins. Primary efficacy end-point is enhanced QMG score and secondary being improved CMAP amplitude.	Significant improvement in both QMG score and CMAP amplitude in 2 trials of 3,4-DAP. IV immunoglobulins, however, did not show significant improvement in CMAP amplitude.
Sanders et al. [29], 2018, North Carolina, USA.	32 patients were randomly selected and the dose of either 3,4-DAP or placebo for more than 3 months was given. >30% deterioration in Triple timed Up-and-Go (3TUG) and changes in self-assessment of LEMS-related weakness was noted.	Participants receiving 3,4-DAP did not have >30% deterioration in (3TUG) with positive changes in W-SAS, contrary to a placebo group.

Other

Guanidine

Guanidine was approved as the first-line agent in the treatment of LEMS but because of multiple side effects, its usage has been limited [30]. Apparently, guanidine functions by increasing the release of acetylcholine after a nerve impulse. It can also slow down muscle cell membrane depolarization and repolarization rates [31]. If amifampridine is not available or tolerated, then guanidine can be advised with or without pyridostigmine in low doses (1000 mg/day) because of its toxicity profile. It is associated with multiple side effects, such as renal toxicity, and gastrointestinal disturbances such as anorexia, diarrhea, gastric irritation, and bone marrow suppression [30].

Pyridostigmine

Pyridostigmine has been advised in the treatment of moderate to severe weakness along with guanidine. It has better tolerance as compared to the rest of the acetylcholinesterase inhibitors. The mechanism of action of pyridostigmine is by increasing the concentration of acetylcholine in the synaptic clefts by the reversible inhibition of cholinesterase activity to improve the parasympathetic tone [30]. The dose recommended for treating LEMS is wide and ranges from 30 mg to 180 mg. The dosing intervals depends upon the side effects and clinical response. The side effects associated with pyridostigmine are mild such as nausea, abdominal cramping, and diarrhea [28].

In patients suffering from refractory weakness and not responding to acetylcholinesterase inhibitors, immunomodulators are used to target the immune system, as it has been central in showing resistance to the treatment.

The following options can also be used in the treatment of LEMS.

Intravenous Immune Globulin

Intravenous immune globulin (IVIG) is considered a first-line treatment option to treat the refractory pattern of weakness seen in patients with LEMS. The mechanism of action of IVIG is unknown, but it is suspected that it works by neutralizing pathogenic autoantibodies and by regulating autoreactive B cells [32]. The total dose of 2 g/kg is effective for the course of two to five days; maintenance therapy for four to 12 weeks with repeat infusion has also been beneficial for patients who initially responded to IVIG treatment. It takes two to four weeks for IVIG to improve weakness, followed by recurrent weakness in the next few weeks [33]. The complications and side effects associated with IVIG are minor and include laboratory changes, rash, headache, and, rarely, deep venous thrombosis [34].

Table 2 summarizes the immunosuppressive agents used in patients suffering from LEMS.

**Table 2:** Immunosuppressive agents DNA: deoxyribonucleic acid; GFR: glomerular filtration rate

Name	Mechanism of Action	Dosage	Side Effects
Prednisone [35].	Decreases migration of polymorphous-lymphocytes, reducing the activity of the lymphatic system to suppress the immune system. Slowing down vascular permeability to reduce inflammation.	The starting dose is 1 mg/kg to 1.5 mg/kg every other day. After improvement, the dose can be tapered based on the clinical situation of the patient.	Hypotension, osteoporosis, infection, shock.
Azathioprine [36].	Halting DNA replication and blocking purine synthesis. The 6-thioguanine nucleotide is involved in causing immunosuppressive and toxic effects.	Starting from 50 mg twice a day and going up to a total dose of 2 to 3 mg/kg/day.	Gastrointestinal side effects such as nausea, vomiting, flu-like illness, malaise, hepatotoxicity.
Mycophenolate [36]	Decrease proliferation of B and T lymphocytes by halting de-novo guanine synthesis.	1000 mg BID	Nausea, diarrhea, and occasionally leukopenia.
Cyclosporine [37].	It works by inhibiting and decreasing the production of interleukin II and resulting in decreased activity of T lymphocytes.	Starting from 100mg divided into two doses and increased up to 3-6mg/kg/day. Given with pyridostigmine if it does not improve symptoms alone.	Decrease GFR, renal toxicity, hypertension, tremor, gingival hyperplasia, nausea, gingival hyperplasia, and flu-like symptoms.

Rituximab

It is recommended for use when other immunosuppressive agents fail to give sufficient clinical response. Trials [38-39] conducted retrospectively showed the efficacy of these substances in patients suffering from LEMS. It is a monoclonal antibody that works by binding to the CD20 receptor and causing the cytotoxicity of B cell lymphocytes, which results in halting the immune response mediated through this pathway. The standard dose is given to LEMS patients at 375 mg/m^2^ once weekly for four weeks and then every month for the next two months [40]. The side effects associated with rituximab include cardiac disorders, such as arrhythmias and acute coronary syndrome, and in other systems, anaphylaxis, allergic reactions, nausea, and vomiting [41].

Plasma Exchange

Patients suffering from the antibody-mediated disorder with a refractory pattern have multiple options, and plasma exchange has shown beneficial effects in them. In patients suffering from LEMS, it has limited advantages, but with concomitant use with another immunosuppressive disorder, it can be beneficial in some patients [42-43]. The recommended protocol for MG is five exchanges over the course of seven to 14 days but no recommendations have been made for LEMS. Therefore, it is suggested to use the same protocol in LEMS, as the pathology is the same between the two disorders [44].

Prognosis

The major determinant of prognosis in LEMS has been the presence of neoplasm. In patients with SCLC associated with LEMS, longer survival has been observed as compared to patients with SCLC but no associated neurological illness [45]. In patients without any associated neoplasm, survival is normal or near-normal, as seen in a study; in 47 patients with LEMS without SCLC, 10 patients died at a mean age of 70 years unrelated to the LEMS. However, two deaths were possibly related to complications of glucocorticoid therapy [46].

Table 3 provides a list of take-home messages.

**Table 3:** Take-home messages

Take-Home Messages
Lambert-Eaton Myasthenic Syndrome is an autoimmune disorder of the neuromuscular junction.
Antibodies are formed against the Voltage-Gated Calcium Channels (VGCC) on presynaptic nerve terminals, thus inhibiting acetylcholine release.
The classic triad presents as muscle weakness, decreased tendon reflexes and autonomic dysfunction.
The investigative tools that can confirm clinical findings are nerve conduction studies, antibody profile, and electromyography.
The treatment options are primarily focused to improve the symptoms, primarily the weakness.
Amifampridine has been recently approved by the FDA for the symptomatic treatment of weakness and it’s the base salt of 3,4-Diaminopyridine (3,4-DAP).
Other treatment options available include guanidine, pyridostigmine, intravenous immune globulin, immunosuppressive agents (Prednisone, Azathioprine, Cyclosporine, Mycophenolate), rituximab, plasma exchange.

## Conclusions

LEMS is a rare autoimmune-mediated neuromuscular disorder caused by a decrease in acetylcholine release from the neuromuscular junction. The prognosis of LEMS depends upon the presence of malignancy, as it can shorten survival in these patients, but near-normal life expectancy has been observed in idiopathic LEMS. The treatment options are primarily focused on the symptomatic improvement of weakness, and these include amifampridine, guanidine, and pyridostigmine. For refractory weakness, patients can go for IVIG, prednisone, or other immunosuppressive agents. Rituximab and plasma exchange have also been proven to be beneficial in some refractory cases.
